# Metagenomic analysis of intertidal hypersaline microbial mats from Elkhorn Slough, California, grown with and without molybdate

**DOI:** 10.1186/s40793-017-0279-6

**Published:** 2017-11-15

**Authors:** Patrik D’haeseleer, Jackson Z. Lee, Leslie Prufert-Bebout, Luke C. Burow, Angela M. Detweiler, Peter K. Weber, Ulas Karaoz, Eoin L. Brodie, Tijana Glavina del Rio, Susannah G. Tringe, Brad M. Bebout, Jennifer Pett-Ridge

**Affiliations:** 1 Lawrence Livermore National Laboratory, Livermore, CA USA; 2 NASA Ames Research Center, Moffett Field, CA USA; 3 Stanford University, Stanford, CA USA; 4 Bay Area Environmental Research Institute, Petaluma, CA USA; 5 Lawrence Berkeley National Laboratory, Berkeley, CA USA; 6 Department of Energy Joint Genome Institute, Walnut Creek, CA USA

**Keywords:** Microbial mats, Hydrogen, Fermentation, Elkhorn slough, Metagenomics

## Abstract

Cyanobacterial mats are laminated microbial ecosystems which occur in highly diverse environments and which may provide a possible model for early life on Earth. Their ability to produce hydrogen also makes them of interest from a biotechnological and bioenergy perspective. Samples of an intertidal microbial mat from the Elkhorn Slough estuary in Monterey Bay, California, were transplanted to a greenhouse at NASA Ames Research Center to study a 24-h diel cycle, in the presence or absence of molybdate (which inhibits biohydrogen consumption by sulfate reducers). Here, we present metagenomic analyses of four samples that will be used as references for future metatranscriptomic analyses of this diel time series.

## Introduction

Microbial mats are amongst the most diverse microbial ecosystems on Earth, inhabiting some of the most inhospitable environments known, including hypersaline, dry, hot, cold, nutrient poor, and high UV environments. Photosynthetic microbial mats found in intertidal environments are stratified microbial communities. Microbial metabolism under anoxic conditions at night results in the generation of significant amounts of H_2_ and organic acids. The high microbial diversity of microbial mats makes possible a highly complex series of metabolic interactions between the microbes, the nature and extent of which are currently under investigation. To address this challenge, we are using a combination of metagenomics, metatranscriptomics, metaproteomics, iTags and naturally collected, as well as culture-based simplified microbial mats to study biogeochemical cycling (H_2_ production, N_2_ fixation, and fermentation) in mats collected from Elkhorn Slough, Monterey Bay, California. We present here the metagenome data, which will be used as a reference for metatranscriptomic analysis in a later paper.

### Site information

**Fig. 1:**
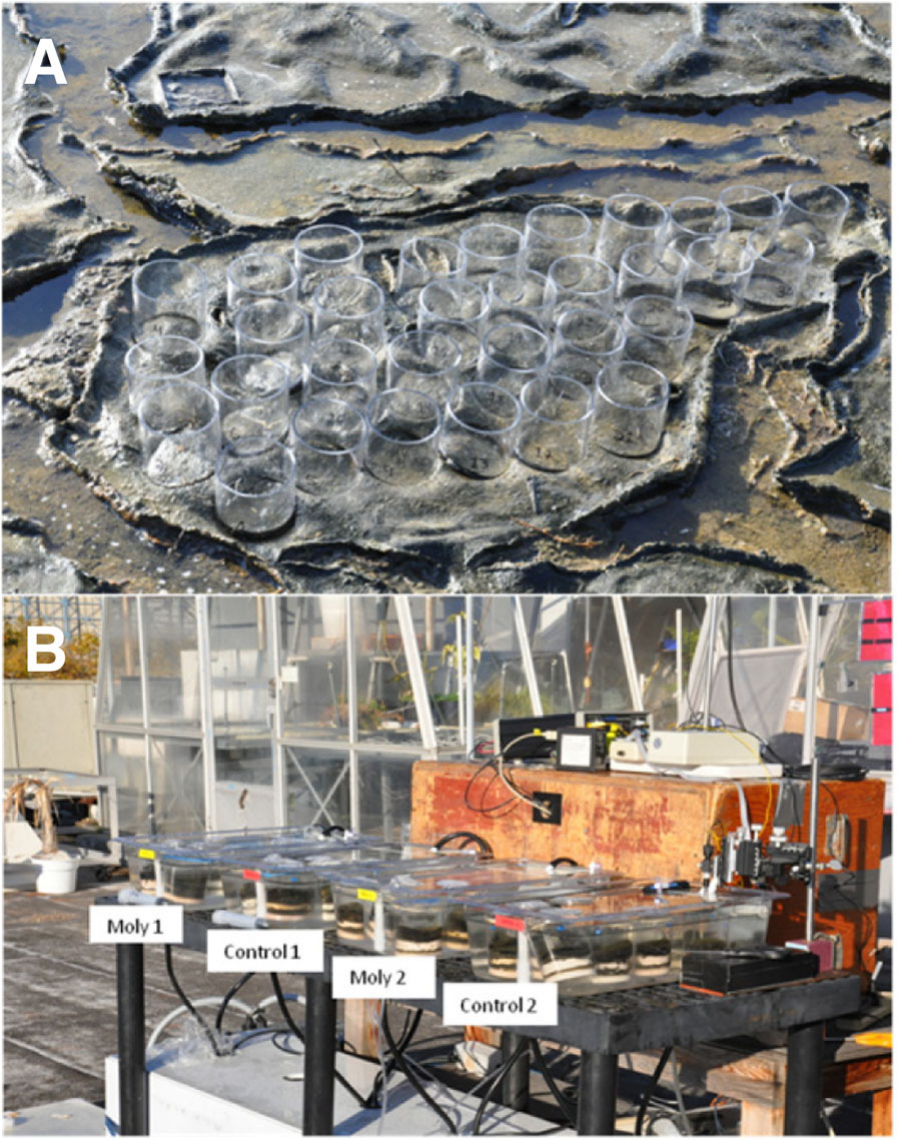
**a**. Photograph of location of cores collected in the field from microbial mats at the Moss Landing Wildlife Area in Elkhorn Slough, Moss Landing, California on 07/11/11. Individual samples collected in core tubes were numbered and could be tracked throughout the diel experiment. **b**. Experimental apparatus used to incubate microbial mats throughout the diel period from 08/11/11 to 09/11/11. Incubation containers containing cores used for control and molybdate treatments are labeled

Cyanobacterial mats are compact, laminated, and highly structured microbial communities (Fig. 1) that comprise great diversity at both the metabolic and phylogenetic level [1] and typically exist in highly saline environments such as lagoons and salterns. These mats notably have a suite of phototrophic organisms and photosynthetic lifestyles, from the dominant cyanobacterial phototroph *Coleofasciculus chthonoplastes* (basionym 10.1601/nm.700 *chthonoplastes*) to purple sulfur and non-sulfur bacteria, and potentially other anoxygenic phototrophs. During the nighttime portion of the diel cycle, phototrophic organisms release fermentation byproducts which in turn help drive a shift from oxic to anoxic metabolism dominated by hydrogen consumption and sulfate reduction by sulfate reducing bacteria such as *Desulfobacteriales* [2]. Naturally occurring mats have a documented capacity to produce and liberate fermentation by-products (H2 and acetate primarily) [3, 4] and to consume them [5, 6] depending on the point in the diel cycle. Lastly, nitrogen assimilation is dominated by nitrogen fixation in these mats, typically by several members of the phylum 10.1601/nm.624 such as ESFC-1 and 10.1601/nm.698 sp. and by sulfate reducing bacteria [7–11]. The mats of Elkhorn Slough are situated in an estuary emptying into Monterey Bay, California and are located in a former salt production pond. The MIMS coding is shown in Table 1.

**Table 1:** Study information

Label	CD2A	CD6A	MD2A	MD6A
IMG/M ID	3,300,000,347	3,300,000,354	3,300,000,919	3,300,000,353
SRA ID	SRX2021703	SRX2021697	SRX2879537	SRX2021699
Study	Gs0067861	Gs0067861	Gs0067861	Gs0067861
GOLD ID (sequencing project)	Gp0053859	Gp0054619	Gp0054089	Gp0054045
GOLD ID (analysis project)	Ga0026496	Ga0026141	Ga0011764	Ga0026171
NCBI BIOPROJECT	PRJNA337838	PRJNA336658	PRJNA366469	PRJNA336698
Relevance	Biotechnological; hydrogen production	Biotechnological; hydrogen production	Biotechnological; hydrogen production	Biotechnological; hydrogen production

Microbial mats like the ones at Elkhorn Slough have long been studied as a model for early life and gained prominence with the discovery that hypersaline mats in Guerrero Negro, Baja California, represented one of the most highly species-diverse microbiomes ever studied [1]. Though not as diverse as the 10.1601/nm.698 mats of the Guerrero Negro system, the Elkhorn Slough mat system captures a similar distribution of organisms observed in laminated seasonal microbial ecosystems [6, 12]. Several areas of microbial mat physiology research are on-going at the Elkhorn Slough site. The site has been used to isolate a novel nitrogen fixer [9] and to show that the majority of fixation is attributable to a 10.1601/nm.698 sp. [10], and to identify the dominant SRB (10.1601/nm.3538) in the ecosystem [2]. Additionally, the site has been investigated for hydrogen cycling. Burow and colleagues [5], showed that hydrogen flux likely originates from the fermentation of photosynthate. This system has also been subjected to metatranscriptomics and metaproteomics analyses [12, 13].

## Metagenome sequencing information

### Metagenome project history

Building on previous work examining gene expression patterns associated with fermentation pathways in microbial mat systems [12], a 24-h study of Elkhorn Slough, CA microbial mats was conducted in 2011. Briefly, field-collected mats were incubated at NASA Ames in seawater media and repeatedly sampled over one diel cycle. In addition, to understand gene expression across the diel cycle, DNA and RNA were extracted from molybdate and control samples for metagenome and metatranscriptome sequencing. Study information is summarized in Table 1.

### Sample information

To understand the variation in gene expression associated with the daytime oxygenic phototrophic and nighttime fermentation regimes in hypersaline microbial mats, a contiguous mat piece was sampled at regular intervals over a 24-h diel period. Additionally, to understand the impact of sulfate reduction on biohydrogen consumption and impacts on community-structure, molybdate was added as an inhibitor to a parallel experiment. Contiguous mat samples were incubated and sampled at regular intervals throughout a 24-h period (8 time points). Four metagenome samples (two time points 12 h apart, from mats with and without molybdate added to the overlying water) and 13 metratranscriptomes (including nine time points for the control time series, four for the molybdate time series, and duplicates for most time points) were sequenced using Illumina technology.

## Sample preparation

Microbial mats used in the experiment were collected using 3 in. acrylic core tubes on the morning of 07/11/11 and transported to Ames Research Center (about one hour by car). The mats were collected from a single contiguous section of mat (Fig. 1a) and were not covered with water at the time of collection (low tide). The microbial mats were immediately transferred to temperature controlled water baths on a rooftop facility [14] (Fig. 1b) containing either seawater or seawater amended with 30 mM (final concentration) sodium molybdate to inhibit the activities of sulfate reducing bacteria. The seawater used was obtained from the boat launch in the Moss Landing harbor at the time of collection of the mats. Two replicate containers each were used for mat incubations: 1) seawater alone and 2) seawater with molybdate water baths.

Mat samples for metagenomic analysis were subsampled from the acrylic core tubes using smaller metal coring tubes (having an area of 1.15 cm2, and a depth of 0.5 cm) on 09/11/11 at 01:30 h and 13:30 h (PST), corresponding to the 2nd and 6th time point in the larger diel time series (one control and one molybdate sample at each time point). Samples were placed in liquid nitrogen immediately after collection and, after flash freezing, were stored in a − 80 °C freezer for later extraction.

The four samples, and resulting metagenomes presented here will be referred to by a 4-character code: CD2A (Control, DNA, time point 2, replicate A), CD6A (Control, DNA, time point 6, replicate A), MD2A (Molybdate, DNA, time point 2, replicate A), MD6A (Molybdate, DNA, time point 6, replicate A). Sample information is provided in Table 2 as per minimal information standards [15].

**Table 2:** Sample information

Label	CD2A	CD6A	MD2A	MD6A
GOLD ID (biosample)	Gb0053859	Gb0054619	Gb0054089	Gb0054045
Biome	Estuarine biome	Estuarine biome	Estuarine biome	Estuarine biome
Feature	Estuarine mud	Estuarine mud	Estuarine mud	Estuarine mud
Material	Microbial mat	Microbial mat	Microbial mat	Microbial mat
Latitude and Longitude	36.812947, −121.784692	36.812947, −121.784692	36.812947, −121.784692	36.812947, −121.784692
Vertical distance	1 m above sea level	1 m above sea level	1 m above sea level	1 m above sea level
Geographic location	Elkhorn Slough, Monterey Bay, California, USA	Elkhorn Slough, Monterey Bay, California, USA	Elkhorn Slough, Monterey Bay, California, USA	Elkhorn Slough, Monterey Bay, California, USA
Collection date and time	09/11/15, 01:30 h (PST)	09/11/15, 01:30 h (PST)	09/11/15, 13:30 h (PST)	09/11/15, 13:30 h (PST)

### DNA extraction

Nucleic acids were extracted from the samples between 2/2/2012 and 24/3/12. For each time point and treatment, the top 2–2.5 mm (photosynthetic layer) of 4 1-cm diameter cores were extracted by initially placing each core in 2 ml tubes containing a mixture of 0.5 ml of RLT buffer (RNeasy Mini Elute Cleanup Kit #74204; Qiagen, Valencia, CA, USA) and 5 μl of 2-mercaptoethanol (cat. # 0482–100) (Amresco, Solon, OH, USA). Samples were homogenized using a rotor-stator homogenizer (Omni International, Kennesaw, GA, USA), followed by the addition of 0.5 mm zirconium beads (OPS Diagnostics, Lebanon, NJ, USA) and then bead-beaten for 40 s using a FastPrep FP120 Cell Disrupter (Qbiogene, Inc., Carlsbad, CA, USA). Samples were spun down and the supernatant for each tube was transferred into a new tube containing an equal volume of phenol:chloroform:isoamyl alcohol (25:24:1) (cat. # 0883–400) (Amresco, Solon, OH, USA). Samples were vortexed, incubated for 5 min at room temperature, and spun down. The supernatant from each tube was transferred to a new tube containing an equal volume of 100% ethanol (Fisher #BP2818, Waltham, MA, USA) and was vortexed. Replicates of supernatant and ethanol mix for each time point and treatment were pooled, run through a QIAmp spin column (QIAmp DNA mini kit #51304, Qiagen, Valencia, CA, USA), and further purified according to the QIAmp DNA mini kit protocol. DNA quality and concentration were measured using a QUBIT fluorometer model Q32857 (Invitrogen, Carlsbad, CA, USA). Samples were submitted to JGI for sequencing.

### Library generation

500 ng of genomic DNA (2 μg for sample MD2A) was sheared using the Covaris E210 (Covaris) and size selected using Agencourt Ampure Beads (Beckman Coulter). The DNA fragments were treated with end repair, A-tailing, and adapter ligation using the TruSeq DNA Sample Prep Kit (Illumina) and purified using Agencourt Ampure Beads (Beckman Coulter). The prepared libraries were quantified using KAPA Biosystem’s next-generation sequencing library qPCR kit and run on a Roche LightCycler 480 real-time PCR instrument. The quantified libraries were then prepared for sequencing on the Illumina HiSeq sequencing platform utilizing a TruSeq paired-end cluster kit, v3, and Illumina’s cBot instrument to generate a clustered flowcell for sequencing. The library information is summarized in Table 3.

**Table 3:** Library information

Label	IUTO	IUTP	HCZO	IUTS
Sample Label(s)	CD2A	CD6A	MD2A	MD6A
Sample prep method	Illumina TruSeq DNA Sample Prep Kit	Illumina TruSeq DNA Sample Prep Kit	Illumina TruSeq DNA Sample Prep Kit	Illumina TruSeq DNA Sample Prep Kit
Library prep method(s)	Illumina TruSeq paired-end cluster kit, v3	Illumina TruSeq paired-end cluster kit, v3	Illumina TruSeq paired-end cluster kit, v3	Illumina TruSeq paired-end cluster kit, v3
Sequencing platform(s)	Illumina HiSeq 2000	Illumina HiSeq 2000	Illumina HiSeq 2000	Illumina HiSeq 2000
Sequencing chemistry	V3 SBS Kit	V3 SBS Kit	V3 SBS Kit	V3 SBS Kit
Sequence size (GBp)	19.6	14.8	13.8	17
Number of reads	130,503,566	98,760,526	91,877,294	113,089,944
Single-read or paired-end sequencing?	Paired-end	Paired-end	Paired-end	Paired-end
Sequencing library insert size	0.27 kb	0.27 kb	0.27 kb	0.27 kb
Average read length	150	150	150	150
Standard deviation for read length	0	0	0	0

### Sequencing technology

Sequencing of the flowcell was performed on the Illumina HiSeq2000 sequencer using a TruSeq SBS sequencing kit 200 cycles, v3, following a 2 × 150 indexed run recipe. All sequencing was performed by the Joint Genome Institute in Walnut Creek, CA, USA.

## Sequence processing, annotation, and data analysis

### Sequence processing

Raw Illumina metagenomic reads were screened against Illumina artifacts with a sliding window with a kmer size of 28, step size of 1. Screened reads were trimmed from both ends using a minimum quality cutoff of 3, reads with 3 or more N’s or with average quality score of less than Q20 were removed. In addition, reads with a minimum sequence length of <50 bps were removed. The sequence processing is summarized in Table 4.

**Table 4:** Sequence processing

Label	IUTO	IUTP	HCZO	IUTS
Tool(s) used for quality control	IMG/M (default)	IMG/M (default)	IMG/M (default)	IMG/M (default)
Number of sequences removed by quality control procedures	5,710,382	4,026,834	2589,674	4,659,580
Number of sequences that passed quality control procedures	124,793,184	94,733,692	89,287,620	108,430,364
Number of artificial duplicate reads	NA	NA	NA	NA

### Metagenome processing

Trimmed, screened, paired-end Illumina reads were assembled using SOAPdenovo v1.05 [16] at a range of Kmers (85, 89, 93, 97, 101, 105). Default settings for all SOAPdenovo assemblies were used (options "-K 81 -p 32 -R -d 1"). Contigs generated by each assembly (6 total contig sets), were de-replicated using in-house Perl scripts. Contigs were then sorted into two pools based on length. Contigs smaller than 1800 bp were assembled using Newbler [17] in attempt to generate larger contigs (flags: -tr, −rip, −mi 98, −ml 80). All assembled contigs larger than 1800 bp, as well as, the contigs generated from the final Newbler run were combined using minimus 2 (flags: -D MINID = 98 -D OVERLAP = 80) [18]. Read depths were estimated based on read mapping with BWA [19]. These sequences are currently available to the public at IMG/M and the JGI genome portals. Metagenome statistics are summarized in Table 5.

**Table 5:** Metagenome statistics

Label	CD2A	CD6A	MD2A	MD6A
Libraries used	IUTO	IUTP	HCZO	IUTS
Assembly tool(s) used	SOAPdenovo v1.05 (default)	SOAPdenovo v1.05 (default)	SOAPdenovo v1.05 (default)	SOAPdenovo v1.05 (default)
Number of contigs after assembly	247,547	141,229	292,231	257,101
Number of singletons after assembly	1,568,087	83,272	1,166,131	1,565,449
minimal contig length	200	200	200	200
Total bases assembled	152,203,650	90,602,774	173,570,670	178,522,206
Contig n50	749	906	695	1.1 kb
% of Sequences assembled	38%	29%	38%	38%
Measure for % assembled	reads mapped to contigs using BWA	reads mapped to contigs using BWA	reads mapped to contigs using BWA	reads mapped to contigs using BWA

### Metagenome annotation

Prior to annotation, all sequences were trimmed to remove low quality regions falling below a minimum quality of Q13, and stretches of undetermined sequences at the ends of contigs were removed. Low complexity regions were masked using the dust algorithm from the NCBI toolkit and very similar sequences (similarity >95%) with identical 5′ pentanucleotides were replaced by one representative, typically the longest, using uclust [20]. The gene prediction pipeline included the detection of non-coding RNA genes (tRNA and rRNA) and CRISPRs, followed by prediction of protein coding genes.

Identification of tRNAs was performed using tRNAScan-SE-1.23 [21]. In case of conflicting predictions, the best scoring predictions were selected. Since the program cannot detect fragmented tRNAs at the end of the sequences, we also checked the last 150 nt of the sequences by comparing these to a database of nt sequences of tRNAs identified in the isolate genomes using blastn [22]. Hits with high similarity were kept; all other parameters were set to default values. Ribosomal RNA genes were predicted using hmmsearch [23] with internally developed models for the three types of RNAs for the domains of life. Identification of CRISPR elements was performed using the programs CRT [24] and PILERCR [25]. The predictions from both programs were concatenated and, in case of overlapping predictions, the shorter prediction was removed.

Identification of protein-coding genes was performed using four different gene calling tools, GeneMark (v. 2.8) [26],Metagene (v. 1.0) [27], Prodigal (V2.50: November, 2010) [28] and FragGenescan (v. 1.16) [29] all of which are ab initio gene prediction programs. We typically followed a majority rule based decision scheme to select the gene calls. When there was a tie, we selected genes based on an order of gene callers determined by runs on simulated metagenomic datasets (Genemark > Prodigal > Metagene > FragGeneScan). At the last step, CDS and other feature predictions were consolidated. The regions identified previously as RNA genes and CRISPRs were preferred over protein-coding genes. Functional prediction followed and involved comparison of predicted protein sequences to the public IMG database using the usearch algorithm [20], the COG database using the NCBI developed PSSMs [30], the Pfam database [31] using hmmsearch. Assignment to KEGG Ortholog protein families was performed using the algorithm described in [32]. Annotation parameters are summarized in Table 6.

**Table 6:** Annotation parameters

Label	CD2A	CD6A	MD2A	MD6A
Annotation system	IMG/M	IMG/M	IMG/M	IMG/M
Gene calling program	FragGeneScan version 1.16, prokaryotic GeneMark.hmm version 2.8, Metagene Annotator version 1.0, Prodigal V2.50: November, 2010	FragGeneScan version 1.16, prokaryotic GeneMark.hmm version 2.8, Metagene Annotator version 1.0, Prodigal V2.50: November, 2010	FragGeneScan version 1.16, prokaryotic GeneMark.hmm version 2.8, Metagene Annotator version 1.0, Prodigal V2.50: November, 2010	FragGeneScan version 1.16, prokaryotic GeneMark.hmm version 2.8, Metagene Annotator version 1.0, Prodigal V2.50: November, 2010
Annotation algorithm				
Database(s) used	IMG, COG, Pfam, KEGG	IMG, COG, Pfam, KEGG	IMG, COG, Pfam, KEGG	IMG, COG, Pfam, KEGG

## Metagenome properties

Metagenomes were sequenced and assembled into 141,229 (CD6A) to 292,231 (MD2A) contigs, covering 90.6 to 173.6Mbp. GC content of the metagenomes ranged from 46% to 52%. These metagenomes include between 206,164 and 399,161 genes each. More than 99% of these are protein coding, and around 40% have some level of function annotation. Metagenome properties are summarized in Table 7.

**Table 7:** Metagenome properties

Label	CD2A	CD6A	MD2A	MD6A
Number of contigs	247,547	141,229	292,231	257,101
GBp	152,203,650	90,602,774	173,570,670	178,522,206
Number of features identified	354,269	206,164	399,161	389,398
CDS	351,921	204,616	396,301	386,642
rRNA	673	577	834	805
others	1675	971	2026	1951
CDSs with COG	156,087	86,041	199,065	173,132
CDSs with Pfam	157,748	88,969	186,210	178,182
CDS with SEED subsystem	NA	NA	NA	NA
Alpha diversity	NA	NA	NA	NA

### Taxonomic diversity

The taxonomic diversity and phylogenetic structure of the metagenomes was determined based on the best BLASTp hits of assembled protein-coding genes with 60% or more identity to protein in the listed phyla, as calculated by the Phylogenetic Distribution of Genes feature in IMG/M. The phylogeny reported is the one in use in IMG/M [33], which uses the phylogeny described as part of the genomic encyclopedia of *Bacteria* and *Archaea* (GEBA) project [34]. Taxonomic composition is summarized in Table 8. Gene copies are estimated based on the number of genes in the assembled metagenome, multiplied by the average read depth of each gene. This provides a better estimate for the total number of reads coming from each taxon, which is proportional to the abundance of those taxa in the microbial mats. Across the assembled metagenomes, the fraction of annotated genes (not accounting for gene copies) that are unassigned at the 60% sequence identity level ranges between 64% and 67%, with 7–13% mapping to phylum 10.1601/nm.7927, 8–13% phylum 10.1601/nm.624, and 9–16% phylum 10.1601/nm.808. However the estimated gene copies show that these samples are in fact dominated by 10.1601/nm.624 sequences (27–49% of estimated gene copies), with smaller contributions from 10.1601/nm.808, 10.1601/nm.7927, and a variety of other bacterial phyla, and only 34–44% unassigned. The majority of cyanobacterial sequences map to 10.1601/nm.701 (19–39% of the total estimated gene copies) and 10.1601/nm.698 *sp.* 10.1601/strainfinder?urlappend=%3Fid%3DPCC+8106 (3.5–5.5% of estimated gene copies). Other individual bacterial species that capture a large fraction of estimated gene copies at 60% identity include 10.1601/nm.1207 *sp. NAP1* (10.1601/nm.809; up to 3.6% in MD6A), 10.1601/nm.2086 (10.1601/nm.2068; up to 3.3% in CD6A), and 10.1601/nm.20022 (10.1601/nm.22750; up to 2% in MD6A).

**Table 8:** Taxonomic composition

Phylum	CD2A	CD6A	MD2A	MD6A
*Cyanobacteria*	2,886,834	1,682,393	1,341,178	1,831,579
*Proteobacteria*	844,689	368,701	757,946	701,003
*Bacteroidetes*	279,447	117,112	512,734	645,277
*Chloroflexi*	11,158	7671	84,811	7443
*Planctomycetes*	32,641	3990	19,619	19,417
*Firmicutes*	14,252	7592	17,425	13,233
*Verrucomicrobia*	10,189	3125	7299	22,666
*Gemmatimonadetes*	13,305	7096	4257	7385
*Chlorobi*	8996	5188	6181	8539
*Actinobacteria*	8964	3794	8707	6873
*Deinococcus-Thermus*	4724	1281	6013	2722
Unassigned	2,133,807	1,191,276	2,206,260	2,140,978

There are noticeable differences in taxonomic composition among the four metagenomes. For example, the molybdate treated samples MD2A and MD6A contain fewer sequences from phylum 10.1601/nm.624 and more from phylum 10.1601/nm.7927 than the control samples. Some of these differences may be due to spatial heterogeneity in the mat from which the samples were collected.

### Functional diversity

The distribution of COG functional categories is very similar between the four genomes (Table 9), with Pearson correlation of the log of the number of genes assigned to each category ranging from 0.986 (CD2A vs. CD6A) to 0.999 (CD2A vs. MD6A), suggesting a broad functional similarity between the samples, despite differences in species composition.

**Table 9:** Functional diversity

COG Category	CD2A	CD6A	MD2A	MD6A
Translation, ribosomal structure and biogenesis	9405	5221	12,469	11,311
RNA processing and modification	74	26	206	39
Transcription	9669	5290	12,476	10,739
Replication, recombination and repair	11,830	6833	14,356	12,322
Chromatin structure and dynamics	107	62	179	101
Cell cycle control, Cell division, chromosome partitioning	1782	988	2408	1907
Nuclear structure	0	1	4	0
Defense mechanisms	3970	2122	4878	4433
Signal transduction mechanisms	13,275	7589	16,709	13,770
Cell wall/membrane biogenesis	11,461	6586	15,115	13,860
Cell motility	3020	1469	3728	2589
Cytoskeleton	48	12	80	27
Extracellular structures	0	0	2	0
Intracellular trafficking and secretion	4536	2401	6057	4509
Posttranslational modification, protein turnover, chaperones	7137	3962	9349	7808
Energy production and conversion	11,737	6252	15,089	12,719
Carbohydrate transport and metabolism	8698	4741	11,199	9685
Amino acid transport and metabolism	14,099	7254	17,462	15,088
Nucleotide transport and metabolism	3830	2069	5089	4469
Coenzyme transport and metabolism	7489	4104	9368	8213
Lipid transport and metabolism	5603	2666	7504	6460
Inorganic ion transport and metabolism	8887	4635	11,353	10,081
Secondary metabolites biosynthesis, transport and catabolism	4011	2040	4818	4185
General function prediction only	20,092	11,257	26,360	22,338
Function unknown	13,560	7933	18,351	15,032
Not in COGs	198,182	120,123	200,096	216,266

## Conclusions

We sequenced and assembled metagenomes for four samples of microbial mat from the Elkhorn Slough estuary in Monterey Bay, California, to be used as reference data for a diel metatranscriptomic study in the presence or absence of molybdate. All four metagenomes were dominated by cyanobacterial sequences, primarily 10.1601/nm.701. Despite some differences in community composition between the four metagenomes (which may be partly due to spatial heterogeneity in the mat), their functional composition in terms of COG functional categories is quite similar.

## Abbreviations

BLAST: Basic local alignment search tool
COG: Clusters of orthologous groups
IMG: Integrated Microbial Genomes
Pfam: Protein families
SRB: Sulfate reducing bacteria
